# Preparation, Characterization and Bioactivities of Strawberry Polysaccharides

**DOI:** 10.3390/foods14071117

**Published:** 2025-03-24

**Authors:** Libo Wang, Yumeng Zhao, Junwen Liu, Ling Zhu, Yanhui Wei, Kun Cheng, Yaqin Xu

**Affiliations:** 1 College of Arts and Sciences, Northeast Agricultural University, Harbin 150030, China; wanglibo@neau.edu.cn (L.W.); m18713135716@163.com (Y.Z.); ljw19981120@163.com (J.L.); zhuling1998@outlook.com (L.Z.); 2 Feixian Forestry Development Center Linyi, Linyi 273400, China; 3 Heilongjiang Province Academy of Agricultural Sciences Institute of Food Processing, Harbin 150086, China; 4 College of Intelligent Systems Science and Engineering, Harbin Engineering University, Harbin 150001, China; weiyanhui@163.com

**Keywords:** strawberry, polysaccharides, ultrasonic degradation, structure, bioactivities

## Abstract

The aim of this research was to characterize the structure, physicochemical properties and anti-complement activities of two strawberry fruit polysaccharides (DSFP-500 and DSFP-700) obtained by ultrasonic degradation. The molecular weight (*M_w_*) of DSFP-500 was 809 kDa and the *M_w_* of DSFP-700 was 791 kDa, obviously lower than the 9479 kDa weight of the native polysaccharide (PSP). DSFP-500 and DSFP-700 were both composed of the same monosaccharides (Man, Rha, Gal, Glc, Gal and Ara) but the molar ratios were different. The two degraded polysaccharides had good thermal stabilities, as well as good water holding capacity (WHC) and oil holding capacity (OHC). The WHCs of DSFP-500 and DSFP-700 were 5.53 ± 0.08 and 5.70 ± 0.03 g water/g, and the OHCs of DSFP-500 and DSFP-700 were 9.34 ± 0.15 and 9.28 ± 0.29 g oil/g. DSFP-500 and DSFP-700 showed strong free radical scavenging activities in vitro; the ABTS^+^• scavenging rates of DSFP-700 and DSFP-500 were 55.97 ± 0.68% and 52.06 ± 0.85% at 4.0 mg/mL, respectively. Moreover, DSFP-500 and DSFP-700 both had anti-complement activities through the classical pathway and the alternative pathway, though DSFP-700 was more effective than DSFP-500. These findings indicated the potentiality of the degraded polysaccharides from strawberry fruits in functional food and medicine development.

## 1. Introduction

In recent years, the natural polysaccharides isolated from plants have been widely used in food and pharmaceutical industries because of their biological activities, non-toxicity and few side effects [1,2]. However, the bioactivities of polysaccharides are influenced by multiple factors including conformation, backbone structure, monosaccharide composition and molecular weight (*M_w_*) [3,4]. For example, macromolecular weight polysaccharides have low solubility in water and difficulty in crossing the cytomembrane barrier, which limits their effective absorption and utilization in the human body. Accordingly, using appropriate methods to degrade polysaccharides can overcome this limitation. The ultrasonic method is a kind of physical method which degrades polysaccharides due to the acoustic cavitation effect. With ultrasonic treatment, the formation and rupture of cavitation bubbles are rapidly induced in the irradiated liquid medium, resulting in irreversible strand breaking [5]. Compared with chemical degradation, ultrasonic degradation is highly efficient, environmentally friendly and easy to operate. Moreover, ultrasonic degradation can maintain the chemical structure of natural polysaccharides and improve their bioactivities [6,7]. For instance, Xiao et al. prepared polysaccharides from *Flammulina velutipes* with low *M_w_s* by ultrasonic treatment, the primary structure and functional groups of which remained unchanged after degradation [8]. Wu et al. found that degraded polysaccharides from *Laminaria japonica* with low *M_w_* contained the same monosaccharide compositions after ultrasonic degradation [9]. Xu et al. extracted the polysaccharides from *Ganoderma lucidum* and found that the degraded polysaccharides using the ultrasonic method had higher biological activities [10]. Li et al. also found that the degraded polysaccharides produced by *Chaetomium globosum* CGMCC 6882 also showed higher antioxidant and antibacterial activities [11]. Therefore, ultrasonic degradation is a superior method for degrading polysaccharides.

Strawberry is an important cultivated berry fruit crop of the *Rosaceae* plants. Strawberry fruits are considered as “functional foods” due to their abundant active compounds, such as polyphenols, polysaccharides, vitamins and anthocyanins [12]. Recently, many publications have confirmed the antioxidant, anti-cancer and anti-fatigue properties of strawberry fruits [13,14]. It is conceivable that polysaccharides should be an essential ingredient of all active components in the *Rosaceae* fruits. Studies have demonstrated that apple polysaccharides have an anti-inflammatory effect and could improve spatial learning and memory impairment in aging mice [15]. Pear polysaccharides also showed immunoenhancing activity in mice [16]. However, there have been few reports on strawberry polysaccharides; Liu and Lin reported that the strawberry polysaccharides had anti-inflammatory and anti-apoptotic activities [14]. Our research team extracted the strawberry polysaccharide and characterized its structure for the first time [17]. Therefore, more attention should be devoted to strawberry polysaccharides, because their virtues have not yet been discovered.

In the present study, two degraded strawberry polysaccharides were gained by ultrasonic degradation, and their physicochemical properties and structure were investigated. The in vitro free radical scavenging activities of polysaccharides were assessed by ABTS^+^• and DPPH• clearance. In addition, the anti-complement activities of the degraded polysaccharides through the classical pathway and alternative pathway were measured. The research conclusion can provide new ideas for the preparation of degraded strawberry polysaccharides, and can supply a foundation for the utilization of strawberry polysaccharides as biomedical preparations.

## 2. Materials and Methods

### 2.1. Materials

The strawberry fruit (Fragaria ananassa Duchesne) was purchased from a professional berry planting cooperative in Shangzhi (Heilongjiang, China). The strawberry fruit was cleaned, vacuum packed and stored in a −20 °C freezer. Sephadex G-200 and 1-phenyl-3-methyl-5-pyrazolone (PMP) were purchased from Shanghai Yuanye Biotechnology Co., Ltd. (Shanghai, China). The 0.5% rabbit red blood cells, 2% sheep red blood cells and the normal healthy adult serum (NHS) were acquired from Hongquan Biotechnology Co., Ltd. (Guangzhou, China). Trifluoroacetic acid (TFA) and trichloroacetic acid (TCA) were bought from Dingguo Changsheng Biotech Co., Ltd. (Beijing, China).

### 2.2. Ultrasonic Degradation of the Native Polysaccharide

The native strawberry fruit polysaccharide (PSP) was prepared in our previous study [17], while ultrasonic treatment was used to prepare degraded polysaccharides in this study. According to the method of Xiao et al. [8], the PSP solution (2.0 mg/mL) was processed by a JY92-2D Ultrasonic cell grinder (Xinzhi Biotechnology Co., Ltd., Ningbo China). The temperature and time were set at 25 ± 1 °C and 80 min, and two different powers (500 and 700 W) were chosen, respectively. The solution obtained after ultrasonic degradation was dialyzed (*M_w_* cut-off 3500 Da) by running water for 48 h and then deionized water for 48 h. After that, the samples were treated by concentration, centrifugation and freeze-drying. Then, they were purified with Sephadex G-200 according to the method in our previous study [18]. The collected eluate was concentrated and lyophilized to gain the high-purity degraded polysaccharides, named DSFP-500 (500 W) and DSFP-700 (700 W), separately.

### 2.3. Determination of Physicochemical Properties of DSFP-500 and DSFP-700

#### 2.3.1. Chemical Composition

The chemical components of DSFP-500 and DSFP-700 including total sugar, reducing sugar, uronic acid, polyphenol and protein were assayed by the phenol–sulfuric acid method [19], 3,5-dinitrosalicylic acid method [20], meta-hydroxydiphenyl method [21], Folin–Ciocalteu method [22] and Bradford method [23], respectively.

#### 2.3.2. Particle Sizes and *M_w_s*

With a scattering angle of 90°, the particle sizes of the DSFP-500 and DSFP-700 solutions (2.0 mg/mL) were measured using the Nano ZS90 particle size and potential analyzer (Malvern Instrument Ltd., Worcestershire, UK) at 25 °C.

Dextran standards (T10, T40, T70, T110, T500, T-2000 and T3755) and the two polysaccharide solutions (1.0 mg/mL) were prepared before analysis. The *M_w_s* of the samples were determined by gel permeation chromatography. The same test process and parameters have been published in our previous study [24].

#### 2.3.3. Monosaccharide Composition

The monosaccharide compositions of DSFP-500 and DSFP-700 were measured based on the reported methods [25]. Trifluoroacetic acid (TFA, 2.0 mol/L, 3.0 mL) was added to the DSFP-500 and DSFP-700 solutions (2.0 mg/L, 5.0 mL) separately and heated at 110 °C for 6 h. After the samples were cooled to 25 °C, 20.0 mL methanol was added in triplicate to remove TFA. The standard monosaccharides (glucose, galactose, arabinose, galacturonic acid, glucuronic acid, mannose, rhamnose) and DSFP-500 and DSFP-700 (10.0 mg) after hydrolysis were mixed with PMP methanol solution (0.3 mol/L, 300 μL) and NaOH solution (0.3 mol/L, 300 μL), then reacted at 65 °C for 1 h. After cooling down, HCl solution (0.3 mol/L, 300 μL) was poured into the resulting samples. Next, the chloroform was added to the sample until the supernatant was clear and transparent. Subsequently, the supernatant was filtered through 0.22 μm and determined by HPLC (WATERS-2695-2414, Waters Technology Co., Ltd., Milford, MA, USA) equipped with a Symmetry C18 column (4.6 × 150 mm) and the Waters 2998 UV detector (Waters Technology Co., Ltd., Milford, MA, USA). The parameters were as follows: the column temperature was 30 °C, the injection quantity was 10 µL and the mobile phase A:B (0.01 mol/L phosphate buffer solution/acetonitrile) = 83:17 (*v*/*v*) with a flow rate of 0.6 mL/min.

### 2.4. Structural Characterization of DSFP-500 and DSFP-700

#### 2.4.1. Ultraviolet-Visible Spectrophotometer (UV-Vis) and Fourier Transform Infrared Spectroscopy (FT-IR)

UV-Vis spectra of the polysaccharide solutions (1.0 mg/mL) were determined on a dual-beam UV-visible spectrophotometer (UV-2700, Shimadzu Corporation, Kyoto, Japan) in the full-wavelength range of 200–900 nm [26].

The FT-IR spectra of DSFP-500 and DSFP-700 were analyzed on an FT-IR spectrometer (ALPHA-T, Bruker Co., Billerica, MA, USA) using KBr pallet within a scope from 4000 to 500 cm^−1^ [18].

#### 2.4.2. Nuclear Magnetic Resonance (NMR)

The DSFP-500 and DSFP-700 samples were dissolved in D_2_O (20.0 mg/mL) and gathered in NMR tubes. The ^1^H NMR, ^13^C NMR, ^1^H/^1^H COSY and ^1^H/^13^C HSQC spectra were measured on a Bruker Av600 MHz spectrometer (AVANCE III 600MHz, Bruker Co., Elmsford, NY, USA).

#### 2.4.3. Congo Red Test

DSFP-500 and DSFP-700 (2.0 mg/mL, 100 μL) were separately mixed with the Congo red reagent (80.0 μmol/L, 2.0 mL) then added to the NaOH solution from 0.0 to 0.5 mol/L and left for 10 min [27]. The solution was scanned using a dual-beam UV-visible spectrophotometer (UV-2700, Shimadzu Corporation, Kyoto, Japan) from 400 to 700 nm to obtain the maximum absorption wavelength (λ_max_). Deionized water was used as a blank control.

#### 2.4.4. X-Ray Diffraction (XRD) and Scanning Electron Microscopy (SEM)

The X-ray diffraction patterns of DSFP-500 and DSFP-700 were obtained by an X’ Pert3 Powder diffractometer (PANalytical B.V., Almelo, The Netherlands). A Cu Ka ray and a scan angle of 5–80° were applied at a scan rate of 5°/min. The voltage was 40 kV and the current was 40 mA.

DSFP-500 and DSFP-700 were coated with a gold film (100 nm thickness) under vacuum, and the morphological characteristics of DSFP-500 and DSFP-700 were confirmed by scanning electron microscope (JSM-6480A SEM, JEOL, Ltd., Tokyo, Japan).

### 2.5. Determination of Functional Properties of DSFP-500 and DSFP-700

#### 2.5.1. Water Holding Capacity (WHC) and Oil Holding Capacity (OHC)

The WHC and OHC of the two polysaccharides were estimated by the method of Wang et al. [28]. DSFP-500 and DSFP-700 solutions (1%, *w*/*v*) were prepared using water and soybean oil as solvents, respectively. The solutions were treated for 30 min at 20–100 °C and centrifuged for 10 min at 4000 r/min. Then, the WHC and OHC were calculated according to the Formula (1).

(1) $$\mathrm{Holding water}\;(\mathrm{oil})\;\mathrm{capacity}=\frac{W_{1}-W_{2}}{W_{0}}\times 100\%$$

where *W*_1_ refers to the total mass of the centrifuge tube before centrifugation (g), *W*_2_ is the remaining mass after separating the supernatant (g) and *W*_0_ is the total mass of the solution (g).

#### 2.5.2. Emulsion Properties

According to the published reference [29], soybean oil (5.0 mL) was mixed with DSFP-500 or DSFP-700 solution (1.0% *w*/*v*, 15.0 mL) at room temperature. Then, the mixture (100 μL) was homogenized at 12,000 rpm for 120 s in a high-speed shear homogenizer (JR500E-SH, Shanghai Jiuran Instrument Equipment, Shanghai, China) and immediately mixed with sodium dodecyl sulfate (0.1%, *w*/*v*, 5.0 mL). The absorbance (*A*_0_) of the emulsion was measured at a wavelength of 500 nm, and the emulsification activity index (EAI) was calculated according to Equation (2). The absorbance (*A*_10_) at 500 nm was tested again after 10 min. The emulsification stability index (ESI) was calculated according to Equation (3).

(2) $$\mathrm{EAI}\left(m^{2}/g\right)=\frac{2\times 2.303\times A_{0}}{\varphi \times m}$$

(3) $$\mathrm{ESI}=\frac{A_{10}}{A_{0}}\times 100\%$$

where φ and *m* were the volume percent of oil (%) and the mass of the sample (g), respectively.

#### 2.5.3. Thermal Stability Analysis

The thermal weight curve (TG) and the differential thermal weight curve (DTG) of DSFP-500 and DSFP-700 were measured by a thermal weight analyzer (STA449, Nichi Scientific Instrument Trading Co., Ltd., Shanghai, China). Measurement conditions: the nitrogen flow rate was 50.0 mL/min, the sample mass was 5.0 mg, the temperature was 30–500 °C and heating rate was 10 °C/min [29].

#### 2.5.4. Rheological Analysis

The rheological properties of DSFP-500 or DSFP-700 were determined. The methods and conditions were completely in accordance with our previous study [17].

### 2.6. Measurement of Free Radical Scavenging Activities of Two Polysaccharides

#### 2.6.1. The ABTS^+^• Scavenging Activity

The ABTS^+^• radical working solution was prepared as follows: Firstly, 2.0 mL 7.4 mmol/L of ABTS solution and 35 μL of potassium persulfate solution (140.0 mmol/L) were mixed evenly. Then, the mixture was placed in the dark for 15 h and diluted with absolute ethanol to determine the absorbance of the mixture at 734 nm. Then, 2.0 mL of ABTS^+^• working solution was poured into the polysaccharide solution (0.1 mL, 0.2, 0.4, 0.6, 0.8, 1.0, 1.0, 1.5, 2.0, 4.0 mg/mL), respectively. After 6 min, the absorbance of the mixture was also measured at 734 nm in triplicate, and ascorbic acid (Vc) was used as a positive control. The clearance of ABTS^+^• was calculated by the Formula (4):

(4) $${\mathrm{ABTS}}^{+}•\;\mathrm{clearance}=(1-\frac{A_{1}-A_{2}}{A_{0}})\times 100\%$$

where *A*_0_ was the absorbance of the ABTS^+^• solution, *A*_1_ was the absorbance of the sample solution and *A*_2_ was the absorbance of the sample solution.

#### 2.6.2. The DPPH• Scavenging Activity

Firstly, 2.0 mL of polysaccharide solution was added in the test tube with a concentration of 0.2, 0.4, 0.6, 0.8, 1.0 and 2.0 mg/mL, then 2.0 mL DPPH• solution was added. The mixed solution was placed in a dark place for 30 min and then the absorbance of the sample was measured at 517 nm. Vc was used as a positive control. The clearance of DPPH• was calculated by the Formula (5):

(5) $$\mathrm{DPPH}•\;\mathrm{clearance}=(1-\frac{A_{2}-A_{1}}{A_{0}})\times 100\%$$

where *A*_1_ was the absorbance of the sample–ethanol solution, *A*_2_ was the absorbance after the mixing reaction between the sample and the DPPH solution and *A*_0_ was the absorbance of the DPPH–ethanol solution.

### 2.7. Measurement of Anti-Complement Activities of Two Degraded Polysaccharides

#### 2.7.1. Anti-Complement Activities Through the Classical Pathway

The anti-complement activities of DSFP-500 and DSFP-700 on the classical pathway were tested according to the previous study [30]. Firstly, 2% sheep red blood cells were diluted with PBS solution to a constant volume until the concentration was 2 *×* 10^9^ cells/mL, then mixed with hemolysin in a 1:1 ratio and incubated at 37 °C for 30 min. Then, the samples were concentrated by a refrigerated centrifuge (4500 rpm) at 4 °C for 10 min to remove unconjugated hemolysin. The NHS was dissolved in PBS (10 mmol/L, pH 7.4) with different concentrations (0.2–4.0 mg/mL), then the degraded polysaccharide solutions were each added. After 30 min of incubation in a 37 °C water bath, 2% sheep erythrocytes were added. The supernatant and the absorbance were determined at 500 nm. A total of 100 μL of 2% sheep red blood cell diluted with 0.5 mL of deionized water was set as a complete control group. The hemolysis rate was calculated based on our reported method [17].

#### 2.7.2. Anti-Complement Activities Through the Alternative Pathway

The anti-complement activities through the alternative pathway were determined using a similar method to that described in Section 2.7.1. The NHS was diluted in PBS (10 mmol/L, pH 7.4)/EDTA buffer using 1:4 by volume. The NHS solution and the polysaccharide solutions with different concentrations were preincubated for 15 min at 37 °C, and then 0.5% rabbit erythrocyte was added for a further 30 min incubation at 37 °C. After centrifugation, the supernatant was placed in a 96 well plate and the absorbance was measured at 405 nm. Heparin was used as the positive control and the rabbit erythrocytes (0.5%, 200.0 μL) were added, with deionized water (300.0 μL) used as the complete control group.

### 2.8. Statistical Analysis

Each experiment was performed in triplicate. The obtained data were rendered as mean ± standard deviation (SD). Difference significance analysis was performed using SPSS 18.0 (SPSS Inc., Chicago, IL, USA); *p* < 0.05 was statistically significant.

## 3. Results and Discussion

### 3.1. Preparation of DSFP-500 and DSFP-700

**Figure 1:**
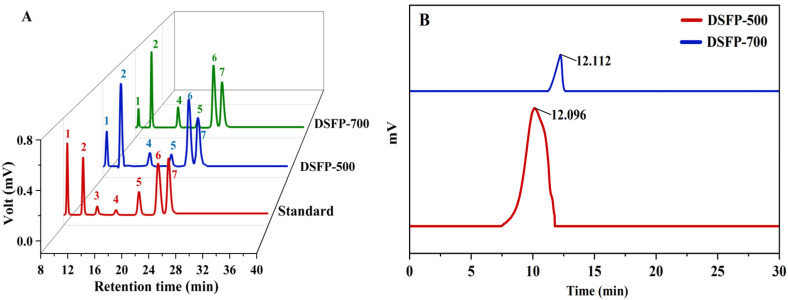
(**A**) Monosaccharides (peak identification: 1, D-mannose; 2, L-rhamnose; 3, D-glucose acid; 4, D-galacturonic acid; 5, D-glucose; 6, D-galactose; 7, L-arabinose) and (**B**) the *M_w_s* of DSFP-500 and DSFP-700.

The native polysaccharide with the *M_w_* of 9479 kDa from strawberry was obtained through hot water extraction and purification with a D4006 macroporous resin column [17]. After ultrasonic treatment at 500 and 700 W, two degraded polysaccharides named DSFP-500 and DSFP-700 were prepared. The chemical compositions of DSFP-500 and DSFP-700 are listed in Table 1. The total sugar of DSFP-500 and DSFP-700 were 86.04 ± 0.12% and 85.25 ± 0.55%, respectively. The uronic acid contents in DSFP-500 and DSFP-700 were 25.30 ± 0.17% and 25.59 ± 0.19%, respectively. Trace quantities of protein and polyphenol were contained in two degraded polysaccharides. DSFP-500 and DSFP-700 were both composed of Man, Rha, GalA, Glc, Gal and Ara with different molar ratios (Figure 1A), which was the same as the monosaccharide composition of the native polysaccharide [17], proving that the ultrasonic treatment was a mild method to degrade polysaccharides without altering the sugar units. The particle size of DSFP-700 (257.3 ± 0.33 nm) was slightly smaller than that of DSFP-500 (270.4 ± 0.42 nm). Based on the retention time, the *M_w_* of DSFP-500 was identified as 809 kDa and the *M_w_* of DSFP-700 was 791 kDa (Figure 1B), compared to 9479 kDa of PSP. The reduction rates of *M_w_* were 91.46% (DSFP-500) and 91.65% (DSFP-700), suggesting a significant degradation effect.

### 3.2. Characterization of DSFP-500 and DSFP-700

#### 3.2.1. UV-Vis and FT-IR Spectra

**Figure 2:**
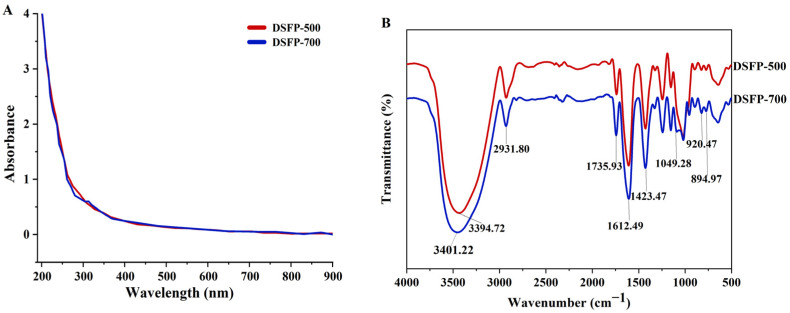
UV-Vis spectra (**A**) and FT-IR spectra (**B**) of the degraded polysaccharides.

As shown in Figure 2A, the UV-Vis spectra of DSFP-500 and DSFP-700 were basically the same; there were no obvious corresponding absorption peak around 260, 280 and 520 nm. The results showed that the degraded polysaccharides did not contain protein, nucleic acid and anthocyanins.

As shown in Figure 2B, DSFP-500 and DSFP-700 displayed the typical absorption peaks of polysaccharides in the range of 4000–500 cm^−1^. The strong absorption peak that appeared at around 3401.22 cm^−1^ was ascribed to the stretching vibrations of O-H [29,31]. The weak peak at 2931.80 cm^−1^ was due to C-H stretching [32]. The bands around 1732.08 and 1612.49 cm^−1^ were attributed to the stretching vibration of -COOR and -COO^−^, respectively [31,33]. The absorption at 1049.28 cm^−1^ proved the existence of a pyranose ring [29,31]. Additionally, the peaks at 894.97 cm^−1^ proved the concurrent existence of α-configurations [29].

#### 3.2.2. NMR Analysis

**Figure 3:**
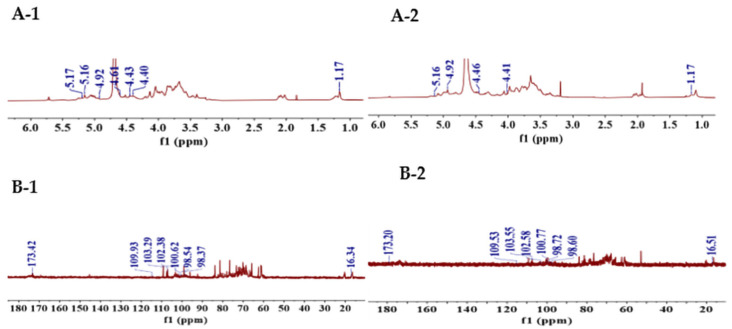

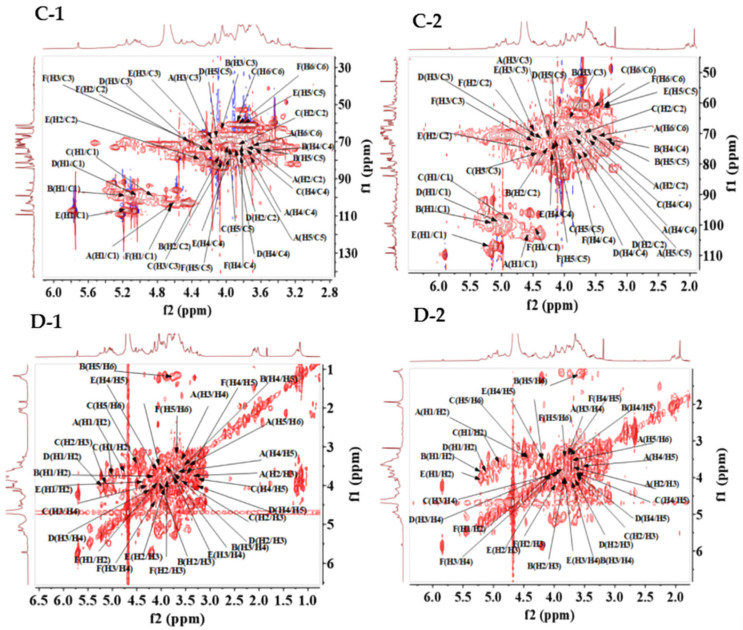
(**A**) ^1^H NMR spectra of DSFP-500 (**A-1**) and DSFP-700 (**A-2**); (**B**) ^13^C NMR spectra of DSFP-500 (**B-1**) and DSFP-700 (**B-2**); (**C**) HSQC of DSFP-500 (**C-1**) and DSFP-700 (**C-2**); (**D**) ^1^H–^1^H COSY of DSFP-500 (**D-1**) and DSFP-700 (**D-2**).

1D and 2D NMR were used to identify the configuration and arrangement of polysaccharides. As shown in Figure 3A,B, the ^1^H and ^13^C NMR spectra of the two degraded polysaccharides exhibited the same characteristic signal peaks, which were mainly distributed in δ_H_ 3.0–5.3 ppm and δ_C_ 60.0–110.0 ppm. Based on the resonance signal region of anomeric protons (δ_H_ 4.5–5.5 ppm) and anomeric carbon (δ_C_ 90.0–110.0 ppm), α-/β-configurations were confirmed [28,34] in degraded polysaccharides. This result agreed with the FT-IR analysis in Section 3.2.1. Based on HSQC spectra (Figure 3C), the chemical shifts in δ5.16/109.93, δ4.43/103.29, δ4.40/102.38, δ4.61/100.62, δ4.92/98.54 and δ5.17/98.37 ppm in DSFP-500, and δ5.16/109.53, δ4.46/103.55, δ4.41/102.58, δ4.66/100.77, δ5.16/98.72 and δ4.92/98.60 ppm in DSFP-700, were assigned to the anomeric region of H1/C1. These data also suggested that both DSFP-500 and DSFP-700 had the same type of sugar residues; this was consistent with the monosaccharide composition results. According to the COSY spectrum (Figure 3D), all the proton chemical shifts (H1 to H6) were claimed, and six sugar residues including → 3,6)-β-D-Galp-(1 → [34], → 2)-α-L-Rhap-(1 → [17,29], → 3)-β-D-Manp-(1 → [18], → 4)-α-D-GalpA-(1 → [29], α-L-Araf-(1 → [30] and → 4)-β-D-Glcp-(1 → [17,18] were identified (as shown in Table 2). The residues in DSFP-500 and DSFP-700 were the same as that of the native polysaccharide [17].

#### 3.2.3. Congo Red Test Analysis

**Figure 4:**
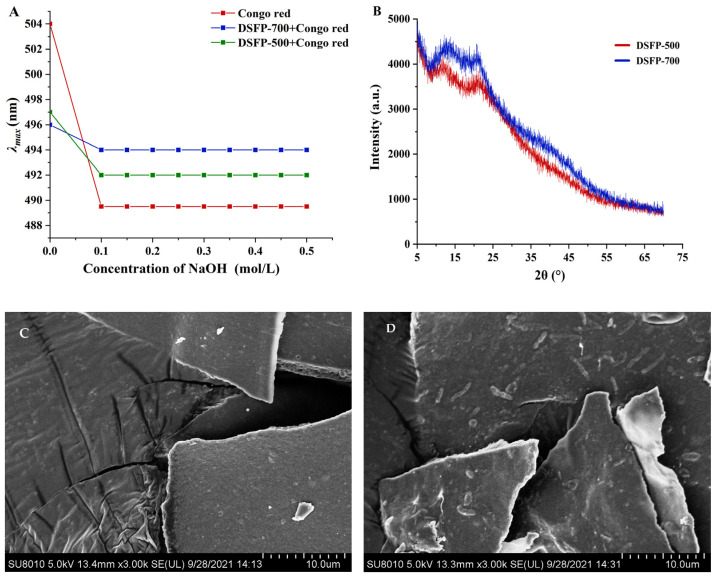
Effect of DSFP-500 and DSFP-700 on the absorbance of Congo red at various NaOH concentrations (**A**); X-ray diffraction of DSFP-500 and DSFP-700 (**B**); SEM images of DSFP-500 (**C**) and DSFP-700 (**D**) (magnification 3000×, scale bar 100 μm).

The Congo red test is commonly used to detect triple helical structures in polysaccharides as Congo red forms a stable complex with the trihelical polysaccharide [34]. As shown in Figure 4A, the maximum absorption wavelength (λ_max_) of DSFP-500 and DSFP-700 did not change in the range of 0 to 0.5 mol/L NaOH; this trend was consistent with the Congo red solution, indicating that neither DSFP-500 nor DSFP-700 had a triple helical conformation, because both DSFP-500 and DSFP-700 were heteropolysaccharides and hardly formed the triple helical conformation [27,34].

#### 3.2.4. XRD and SEM Analysis

Through X-ray diffraction technology, the crystal cell and lattice composition of the substance can be measured accurately [35], so as to better understand the crystal morphology of the polysaccharides. Figure 4B showed the degree of crystallization of DSFP-500 and DSFP-700. At the angles of 5–70°, two polysaccharides did not exhibit particularly obvious diffraction peaks, and only small diffusion diffraction peaks appeared at about 20°. These data showed that both DSFP-500 and DSFP-700 were non-crystalline substances [9,36].

SEM is an effective method to survey the morphology of polysaccharides [37]. As shown in Figure 4C,D, both DSFP-500 and DSFP-700 presented irregular sheet structures, but DSFP-700 had more small fragments than DSFP-500. The reason for this phenomenon might be that the native polysaccharide was decomposed and the glycoside bonds of the polysaccharides were broken under high intensity ultrasound, so the fragments could not form a larger sheet structure [9,36]. Xu et al. prepared degraded polysaccharides from *Ganoderma lucidum* and scanned them for their microstructures. The results indicated that their surface areas were decreased significantly compared to the native polysaccharides, which might be due to the breakage of glycoside bonds between the polysaccharides caused by ultrasonic degradation [10].

### 3.3. Functional Properties of DSFP-500 and DSFP-700

#### 3.3.1. WHC and OHC

WHC represents the water retention capacity of the sample and is usually used to judge the stabilizing effect and sensibility of the sample [38,39], and OHC can reflect the ability of substances to adsorb oil, with a high OHC content contributing to the stability of high-fat foods [40]. The results of the experiments indicated that the WHCs of DSFP-500 and DSFP-700 were 5.53 ± 0.08 and 5.70 ± 0.03 g water/g and the OHCs of DSFP-500 and DSFP-700 were 9.34 ± 0.15 and 9.28 ± 0.29 g oil/g, respectively. They were both better than the gluten (1.40 ± 0.05 g water/g and 1.19 ± 0.13 g oil/g). The reason for this might be that the ultrasonic method significantly changed the pore structure of the polysaccharides and released a large number of polar groups, thus greatly enhancing their water holding capacity [39]. Gluten has a significant effect in food industries due to its good functional characteristics. Therefore, DSFP-500 and DSFP-700 may be used as stabilizers in roasted foods and as hydrophilic surfactants.

#### 3.3.2. Emulsification

Based on their polymer structures, polysaccharides are the most promising emulsifying agents in medicine, food and other industries [2,4]. The EAI of DSFP-500 and DSFP-700 (1.0%, *w*/*v*) reached 80.26 ± 3.58 and 91.02 ± 2.19 m^2^/g, respectively. Moreover, the ESIs of DSFP-500 and DSFP-700 were 66.17 ± 2.06 and 78.27 ± 2.07 min. Under the same conditions, the EAI and ESI of soy lecithin were 127.09 ± 0.69 m^2^/g and 93.02 ± 1.77 min, respectively. It is well known that soy lecithin is an emulsifier used in the food, cosmetic and pharmaceutical industries [40]; therefore, DSFP-500 and DSFP-700 also have the potential to be used as an emulsifier.

#### 3.3.3. Thermal Analysis

**Figure 5:**
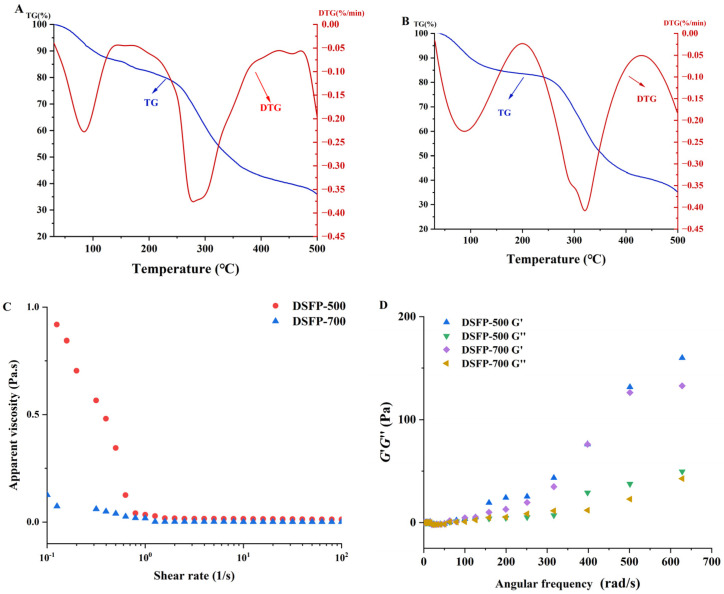
TG and DTG curves of DSFP-500 (**A**) and DSFP-700 (**B**); rheological behaviors of DSFP-500 and DSFP-700; (**C**) steady shear flow curve and (**D**) *G*′ and *G*″ with angular frequency.

Heating stability is an important characteristic of biological applications, which can quantitatively measure the variation in material mass over time and the temperature during dehydration, decomposition and oxidation processes [28,29]. The thermal stabilities of DSFP-500 and DSFP-700 were investigated by TG and DTG analysis. As shown in Figure 5, both DSFP-500 (Figure 5A) and DSFP-700 (Figure 5B) had a three-step decomposition mode, and the first weight loss was about 10% in the range from 30 to 105 °C (decomposition temperature). This stage may be mainly caused by the loss of free water and bound water in the samples. In the second phase, when the temperature increased from the decomposition temperature to about 400 °C, the DTG curve showed that the accelerated heat loss rate and the maximum weight loss rate occurred accordingly. The polysaccharide weight also began to decrease significantly (TG curve). Moreover, the weight loss of DSFP-500 (47.60%) and 47.23% of DSFP-700 suggested the depolymerization and decomposition of polysaccharides [34]. With the temperature increasing from 400 to 500 °C, the thermal decomposition rate of the two samples slowed down, and the residual masses of strawberry polysaccharides were both 35.92%. Compared with the 26.73% residual mass of the native polysaccharide [17], the two degraded polysaccharides had better thermal stabilities.

#### 3.3.4. Rheological Behaviors

As shown in Figure 5C,D, with the increase in shear rate, DSFP-500 and DSFP-700 exhibited non-Newton rheological behaviors with shear thinning. The *G*′ and *G*″ of DSFP-500 and DSFP-700 were both dependent on the frequency; the values of *G′* were higher than the values of *G*″ in the tested frequency range. In the same conditions, the *G*′ and *G*″ values of DSFP-500 were both higher than that of DSFP-700. These results proved that DSFP-500 possessed better gelling properties than DSFP-700, but that DSFP-500 and DSFP-700 both showed weaker gel behavior compared to PSP [17].

### 3.4. Free Radical Scavenging Activities of DSFP-500 and DSFP-700

#### 3.4.1. The Scavenging Activity on ABTS^+^•

**Figure 6:**
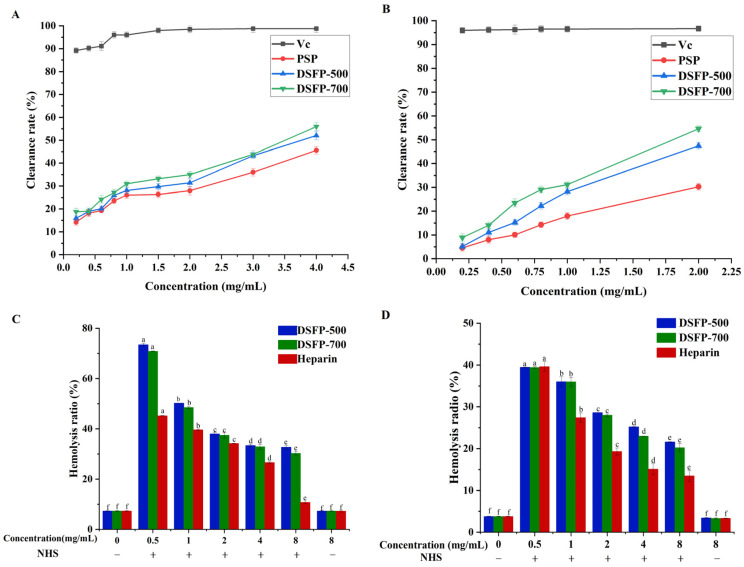
Scavenging activities of two degraded polysaccharides on ABTS^+^• (**A**) and DPPH• (**B**). Hemolysis ratio of two degraded polysaccharides through classic pathway (**C**) and alternative pathway (**D**). Values with different letters in columns represent differences (*p* < 0.05).

ABTS can react with potassium persulfate to produce ABTS^+^•, which has a maximum absorption wavelength at 734 nm with a blue–green color. However, antioxidants can inhibit the formation of ABTS^+^•, thus fading the solution [9,11]. The scavenging activities of the three polysaccharides against ABTS^+^• were determined. As shown in Figure 6A, the clearance capacity of Vc for ABTS^+^• remained high in the range of 0.2–4.0 mg/mL. Under the same concentrations, the two degraded polysaccharides showed similar scavenging activities, both of which were higher than that of PSP. At 4.0 mg/mL, the scavenging rates of Vc, DSFP-700, DSFP-500 and PSP were 98.77 ± 0.65%, 55.97 ± 0.68%, 52.06 ± 0.85% and 45.59 ± 0.63%, respectively. It can be seen that the in vitro free radical scavenging activity of degraded polysaccharides is significantly higher than that of natural polysaccharides (*p* < 0.05), and that DSFP-700 with lower *M_w_* exhibited an even higher free radical scavenging activity.

#### 3.4.2. The Scavenging Activities on DPPH•

DPPH• can supply hydrogen through antioxidants, forming stable DPPH• molecules, and thus is often used to assess the free radical scavenging capacity of antioxidants. As shown in Figure 6B, both of the degraded polysaccharides showed good DPPH• clearance abilities with a certain concentration dependence (*p* < 0.05). Under the tested conditions, the scavenging activities were Vc > DSFP-700 > DSFP-500 > PSP. When the concentration of 2.0 mg/mL was used, the scavenging rates were 96.66 ± 0.26%, 54.62 ± 0.10%, 47.41 ± 0.45% and 30.26 ± 0.20%, respectively.

It was clear that the radical scavenging capacities of the two degraded polysaccharides were obviously better than that of the native polysaccharide, because more active chemical groups were exposed in the polysaccharides with lower *M_w_*, and the free radicals were more likely to react with the chemical groups [6,7].

### 3.5. Anti-Complement Activities of DSFP-500 and DSFP-700

The complement system is composed of a battery of proteins, which plays an important part in immunoregulation. If this system is activated, the red blood cells rupture and the hemoglobin is released. Therefore, the hemolysis rate is an index of the complement activation level [30,41]. Complement activation can occur through the classical pathway or the alternative pathway; the anti-complement activities of DSFP-500 and DSFP-700 were both determined through these two pathways.

#### 3.5.1. Anti-Complement Activity Through the Classical Pathway

In the complement test (Figure 6C), when the concentration of DSFP-500, DSFP-700 and heparin increased from 0.5 to 8.0 mg/mL, the hemolysis rate of sheep red blood cells decreased (*p* < 0.05), which showed that the presence of the polysaccharides effectively inhibited red blood cell hemolysis and complement system activation. This result was in accord with the study of Jiao et al.: with the increase in the polysaccharide concentration, the anti-complement activity was more obvious [42]. At a concentration of 8.0 mg/mL, the hemolysis rate descended to 32.68 ± 0.80% (DSFP-500), 30.20 ± 0.67% (DSFP-700) and 10.72 ± 0.43% (heparin). When a 50% hemolysis inhibitory rate was reached, the required concentrations of the samples were 1.44 ± 0.17 mg/mL (DSFP-500), 1.28 ± 0.02 mg/mL (DSFP-700) and 0.46 ± 0.15 mg/mL (heparin). It was clear that DSFP-700 had a higher activity than DSFP-500. Zhang et al. obtained degraded sulfated polysaccharides from red alga *Pyropia haitanensis* (LP-G2), and the anti-complement activity of the polysaccharides was analyzed through the classical pathway. When a 50% hemolysis inhibitory rate was reached, the required concentrations of LP-G2 were 3.08 ± 0.25 mg/mL [41]. Apparently, the degraded strawberry polysaccharides showed a higher anti-complement activity.

#### 3.5.2. Anti-Complement Activity Through the Alternative Pathway

Through the bypass pathway, the anti-complement activity of the degraded polysaccharides was evaluated by measuring the hemolysis rate of rabbit red blood cells. As can be seen from Figure 6D, with the increase in DSFP-500, DSFP-700 and with heparin addition, the cell hemolysis rate decreased significantly (*p* < 0.05), suggesting that DSFP-500 and DSFP-700 could inhibit the activation. During the tested concentrations, the hemolysis rates treated with the polysaccharides and heparin were basically consistent with the untreated control group, indicating that the polysaccharides and heparin were not toxic to the cells. When the concentration was 8.0 mg/mL, the hemolysis rates were 21.59 ± 0.05% (DSFP-500), 20.21 ± 0.07% (DSFP-700) and 13.45 ± 0.19% (heparin). DSFP-700 had higher activity than DSFP-500 through the alternative pathway. However, in recent reports on anti-complement activity, most of the polysaccharides often exhibited anti-complement activity only through the classical pathway. For example, Xia et al. found the polysaccharides from *Juniperus pingii var* had a favorable anti-complement activity through the classical pathway, but no inhibiting effects through the alternative pathway [30]. Even if the polysaccharides exhibited anti-complement activity through the alternative pathway, the activity was low. For instance, Kim extracted the crude polysaccharides from Trifoliate Orange (Poncirus trifoliate) seeds named TSCP and determined the anti-complement activity through the alternative pathway. The hemolysis rate was 38.64% at 1.0 mg/mL of TSCP [43], which was higher than 36.01 ± 1.15% (DSFP-500) and 35.97 ± 1.15% (DSFP-700). These results indicated that DSFP-500 and DSFP-700 had better anti-complement activities compared with many plant polysaccharides.

The above experimental results demonstrated that DSFP-700 had greater free radical scavenging activity and anti-complement activity than DSFP-500. The most likely reason for this was that the *M_w_* of DSFP-700 was lower than that of DSFP-500 and the uronic acid content of DSFP-700 was higher than that of DSFP-500. Previous studies have confirmed that these two factors are closely related to the polysaccharide activities [44,45]. There may be other influencing factors, such as the proportion of the monosaccharide composition, viscosity and solubility, but it is not easy to clarify the mechanism based on the current results.

## 4. Conclusions

In the present study, the physicochemical properties, structural characteristics and anti-complement activities of two degraded polysaccharides were investigated. The results showed that DSFP-500 and DSFP-700 had higher activities than the native polysaccharide. In addition, DSFP-700 with lower *M_w_* possessed better physicochemical and functional properties than DSFP-500. DSFP-500 and DSFP-700 were composed of the same monosaccharides as the native polysaccharide, confirming that ultrasonic degradation maintained the main structure and was an effective method to prepare degraded polysaccharides. The current study also showed that two degraded strawberry fruit polysaccharides exhibited strong anti-complement activities. These findings will expand people’s cognition of the functional characteristics of strawberry polysaccharides and will lay the foundation for further research on strawberry use in health foods.

## Figures and Tables

**Table 1:** Chemical compositions of DSFP-500 and DSFP-700.

Chemical Composition	Samples	
	DSFP-500	DSFP-700
Total sugar (%)	86.04 ± 0.12	85.25 ± 0.55
Reducing sugar (%)	0	0
Uronic acid (%)	25.30 ± 0.17	25.59 ± 0.19
Protein (%)	0.17 ± 0.06	0.15 ± 0.08
Polyphenol (%)	0.13 ± 0.08	0.10 ± 0.04
Particle size (nm)	270.4 ± 0.42	257.3 ± 0.33
*M_w_* (kDa)	809	791
Monosaccharide composition (molar ratio)		
Man	1.00	1.00
Rha	2.82	4.37
GalA	2.79	4.61
Glc	1.06	1.06
Gal	1.51	3.30
Ara	1.56	3.36

**Table 2:** The chemical shifts of ^1^H and ^13^C NMR in DSFP-500 and DSFP-700.

Sugar Residues		Chemical Shifts of ^1^H/^13^C (ppm)					
		H1/C1	H2/C2	H3/C3	H4/C4	H5/C5	H6/C6
A →3,6)-β-D-Gal*p*-(1→	DSFP-500	4.43/103.29	3.57/72.71	3.86/75.95	3.65/72.17	3.67/75.23	3.46/69.80
	DSFP-700	4.46/103.55	3.54/72.60	3.85/76.62	3.65/71.06	3.68/75.25	3.50/69.40
B →2)-α-L-Rha*p*-(1→	DSFP-500	5.17/98.37	4.00/83.05	3.87/73.25	3.65/71.05	3.45/70.43	1.17/16.34
	DSFP-700	5.16/98.72	4.05/82.88	3.83/73.29	3.65/71.15	3.48/70.47	1.17/16.51
C →3)-β-D-Man*p*-(1→	DSFP-500	4.61/100.62	3.75/69.41	4.02/80.32	3.62/71.34	3.94/72.37	3.73/61.55
	DSFP-700	4.66/100.77	3.72/69.21	4.04/80.51	3.64/71.48	3.96/72.53	3.74/61.26
D →4)-α-D-Gal*p*A-(1→	DSFP-500	4.92/98.54	3.71/72.37	4.04/75.71	3.64/78.48	3.93/69.07	/173.42
	DSFP-700	4.92/98.60	3.72/72.68	4.01/75.67	3.68/78.55	3.94/69.14	/173.20
E α-L-Ara*f*-(1→	DSFP-500	5.16/109.93	4.15/81.21	3.85/76.53	4.01/81.32	3.65/61.36	–/–
	DSFP-700	5.16/109.53	4.17/81.35	3.86/76.38	4.03/81.27	3.64/61.52	–/–
F →4)-β-D-Glc*p*-(1→	DSFP-500	4.40/102.38	4.16/74.12	4.09/75.20	3.78/79.80	3.87/73.70	3.74/61.87
	DSFP-700	4.41/102.58	4.14/74.11	4.11/75.34	3.76/79.92	3.89/73.42	3.73/61.59

## Data Availability

The original contributions presented in this study are included in the article. Further inquiries can be directed to the corresponding authors.
